# ALPD-Net: a wild licorice detection network based on UAV imagery

**DOI:** 10.3389/fpls.2025.1617997

**Published:** 2025-07-22

**Authors:** Jing Yang, Huaibin Qin, Jianguo Dai, Guoshun Zhang, Miaomiao Xu, Yuan Qin, Jinglong Liu

**Affiliations:** College of Information Science and Technology, Shihezi University, Shihezi, China

**Keywords:** UAV imagery, licorice detection, background suppression, feature fusion, deep learning

## Abstract

**Introduction:**

Licorice has significant medicinal and ecological importance. However, prolonged overharvesting has resulted in twofold damage to wild licorice resources and the ecological environment. Thus, precisely determining the distribution and growth condition of wild licorice is critical. Traditional licorice resource survey methods are unsuitable for complex terrain and do not meet the requirements of large-scale monitoring.

**Methods:**

In order to solve this problem, this study constructs a new dataset of wild licorice that was gathered using Unmanned Aerial Vehicle (UAV) and proposes a novel detection network named ALPD-Net for identifying wild licorice. To improve the model’s performance in complex backgrounds, an Adaptive Background Suppression Module (ABSM) was designed. Through adaptive channel space and positional encoding, background interference is effectively suppressed. Additionally, to enhance the model’s attention to licorice at different scales, a Lightweight Multi-Scale Module (LMSM) using multi-scale dilated convolution is introduced, significantly reducing the probability of missed detections. At the same time, a Progressive Feature Fusion Module (PFFM) is developed, where a weighted self-attention fusion strategy is employed to effectively merge detailed and semantic information from adjacent layers, thereby preventing information loss or mismatches.

**Results and discussion:**

The experimental results show that ALPD-Net achieves good detection accuracy in wild licorice identification, with precision 73.3%, recall 76.1%, and mean Average Precision at IoU=0.50 (mAP50) of 79.5%. Further comparisons with mainstream object detection models show that ALPD-Net not only provides higher detection accuracy for wild licorice, but also dramatically reduces missed and false detections. These features make ALPD-Net a potential option for large-scale surveys and monitoring of wild licorice resources using UAV remote sensing.

## 1 Introduction

Licorice is known as the ‘National Herb’ in China and is a perennial herb of the legume family, genus Glycyrrhiza, primarily growing in regions such as Xinjiang, Inner Mongolia, Gansu, and Ningxia. It has wide medicinal and ecological value (Ding et al., 2022; Dang et al., 2024; Khaitov et al., 2022). There are many species of licorice, among which Glabrous licorice, Spreading fruit licorice, and Ural licorice are the original plants of traditional Chinese medicinal licorice (Jiang et al., 2020). Due to the enormous market demand, wild licorice has been over-harvested for a long time, leading to the dual destruction of wild licorice resources and the ecological environment (Yan et al., 2023). The traditional survey methods are mainly used to determine the distribution of wild licorice resources through a combination of walk-through surveys and sample surveys (Li et al., 2024a), and use sample plant methods or projection coverage methods to assess the reserves of wild medicinal plants. This method is well established but has poor applicability in areas with complex ecological environments. Wild licorice typically grows in arid sandy lands, riverbank sandy soils, hillside grasslands, and salinized soils, and only professionals can carefully distinguish licorice species. As a result, the survey work on wild licorice faces issues such as slow progress, long cycles, and difficulty achieving full coverage (Pohjanmies et al., 2021).

In recent years, multi-level remote sensing technology assisted by Unmanned Aerial Vehicle (UAV) lowaltitude remote sensing has provided a new approach for the investigation of licorice resources (Wongsuk et al., 2024). UAV remote sensing has good mobility and strong timeliness, allowing for more efficient and flexible acquisition of near-surface remote sensing images (Al-lQubaydhi et al., 2024). Since UAV technology enables large-scale operations at a low cost, it finds widespread applications in resource surveys (Williams, 2024) and automatic species identification (Feng et al., 2024). At the same time, deep learning gradually develops and provides a technical foundation for resource surveys, making field resource surveys more efficient and accurate. Ding et al. (2023) used UAV and deep learning technology to detect and assess the yield of wild medicinal plants. They combine ResNet101 and Mask R-CNN to design a detection model, enabling the accurate detection and monitoring management of the Lamioplomis rotata Kudo medicinal plant population. Wang et al. (2024c) used YOLOv7 and YOLOv5 models to train on field UAV images of G. szechenyii flower and G. veitchiorum flower, achieving accurate identification and yield statistics for the flowers of these two wild medicinal plants. Drawing on the ideas of the outstanding researchers mentioned above, this paper combines deep learning object detection technology with UAV remote sensing images to provide a solution for the survey and monitoring of wild licorice resources.

Deep learning-based object detection algorithms can be divided into two categories: two-stage algorithms and single-stage algorithms (Kaur and Singh, 2024). Two-stage algorithms primarily consist of two main phases: candidate box generation and object classification and localization (Manakitsa et al., 2024; Rostami et al., 2024). This two-stage separation design makes the model more flexible and scalable, and it performs well in target localization for small objects and complex scenes (Fan et al., 2024). However, the generation of a large number of candidate boxes results in higher computational complexity and slower detection speed (Li et al., 2024c; Cai et al., 2024). Traditional two-stage detection frameworks, such as R-CNN, Fast R-CNN, and Faster R-CNN (Ren et al., 2017), are characterized by their sequential process of generating region proposals followed by classification and bounding box refinement. In contrast, single-stage algorithms directly predict both the object category and bounding box during the forward pass, without the need for generating candidate regions (Hou et al., 2022). Since single-stage algorithms directly predict the object boxes on each cell or anchor box, they may fail to accurately capture the location information of the object in complex scenes, leading to relatively low target localization accuracy. Additionally, their receptive field is relatively large, which results in poor detection performance for small-sized objects and an increased likelihood of missing detections (Wang et al., 2023, 2024e). However, Single-stage algorithms offer faster detection speeds, making them ideal for applications with strict real-time constraints (Yang et al., 2024b). Popular single-stage object detection models include You Only Look Once (YOLO) (Jiang et al., 2022), Single-Shot Detector (SSD) (Zhai et al., 2020), and others.

Due to the high inference speed, short model training time, and high accuracy of single-stage algorithms, which significantly reduce computation time and cost, they are suitable for rapid detection of large-scale wild licorice resources. Therefore, this paper adopts a single-stage detection algorithm. Compared to other networks, the residual structure of the ResNet network outperforms other traditional deep convolutional neural networks in recognizing objects in complex backgrounds (Cheng et al., 2017; Freitas et al., 2022). It extracts key features from complex data through multi-level nonlinear transformations (Genze et al., 2022), which helps to compensate for the shortcomings of single-stage algorithms in complex scenarios. Considering the impact of network depth and width on detection accuracy and speed, and balancing both factors, the ResNet34 network combined with the decoupled detection head of the single-stage detection algorithm is selected as the base model for this study, referred to as ResNet34-D.

There is a wide variety of weeds in the wild, which shade each other from licorice, and the complex background resulting poses a great challenge for licorice detection (Wang et al., 2021). Fang et al. (2022) proposed a new network architecture, HCA-MFFNet, aimed at addressing the issue of corn leaf disease recognition under complex backgrounds. The network extracted features from corn leaf disease images by applying a Hard Coordination Attention (HCA) mechanism at different spatial scales, thereby reducing the interference of complex backgrounds on recognition. However, HCA-MFFNet lacks the ability to extract detailed target information in more complex backgrounds, which may lead to false detections. The contextual information helped the model better distinguish between objects and backgrounds. Xi et al. (2022) introduced a module that could simultaneously capture both local and global information of the target, and adaptively combine this information to enhance object detection in complex backgrounds. However, feature maps in the network contain redundant background information, resulting in higher computational overhead for the network. The above researchers optimize the model structure for complex background issues, but most of the detections are performed on a single complex background with less background interference. When faced with more complex wild backgrounds, there may be issues with weak extraction of target detail information and information redundancy, which affects the model’s computational speed.

Under different soil environments and climatic conditions, the growth scale of licorice may vary significantly due to factors such as temperature, humidity, and soil composition. Due to the differences in the representation of objects with different scales in the feature layer, the model may not be able to simultaneously capture the key information of both small-scale and large-scale targets (Wu et al., 2025). Therefore, in wild licorice detection, smaller licorice may be overlooked due to blurred features, while larger licorice may lead to overly concentrated features, making it difficult to accurately distinguish the details of the target. This can result in false and missed detection, thus reducing the detection accuracy of licorice (Ren et al., 2024). To address the multi-scale issue, Li et al. (2024b) designed the multi-scale feature selection block, used varying receptive fields to extract rich multi-scale features, and effectively fused features of different scales by adaptively adjusting the receptive field size, significantly improving the network’s recognition ability. Jiang et al. (2024) designed the multiscale feature extraction module, extracted rich and valuable multi-scale feature information by performing convolutions of different scales on multiple branches. Although the model’s detection accuracy improves, this module requires a large number of parameters, which reduces the model’s detection efficiency.

Based on the above issues, this study proposes a wild licorice detection model, ALPD-Net, to achieve efficient and accurate detection of Glycyrrhiza uralensis (G. uralensis), Glycyrrhiza glabra (G. glabra), and Glycyrrhiza inflata (G. inflata) in wild scenes. The contributions of this paper are as follows:

1. The Adaptive Background Suppression Module (ABSM): To enhance the model’s ability to capture detail features of licorice in more complex wild backgrounds, the multi-head self-attention mechanism is combined with spatial coordinate feature encoding. This approach removes redundant information while suppressing interference from complex background information, thereby improving the model’s detection accuracy.
2. The Lightweight Multi-Scale Module (LMSM): LMSM is designed to address the issue of poor model performance when there are significant scale differences in licorice. It uses fewer computations to enhance the model’s ability to recognize multi-scale licorice.
3. The Progressive Feature Fusion Module (PFFM): PFFM is designed to enhance the model’s capability to differentiate between various types of licorice and weeds. It gradually integrates features using Weighted Self-Attention Fusion (WSAF), thereby further enhancing the overall performance of the model.
4. A brand-new wild licorice dataset was constructed, on which ALPD-Net achieved a mAP50 of 79.5%. Compared to other mainstream detection models, ALPD-Net demonstrated superior detection performance.

## 2 Materials and methods

**Figure 1:**
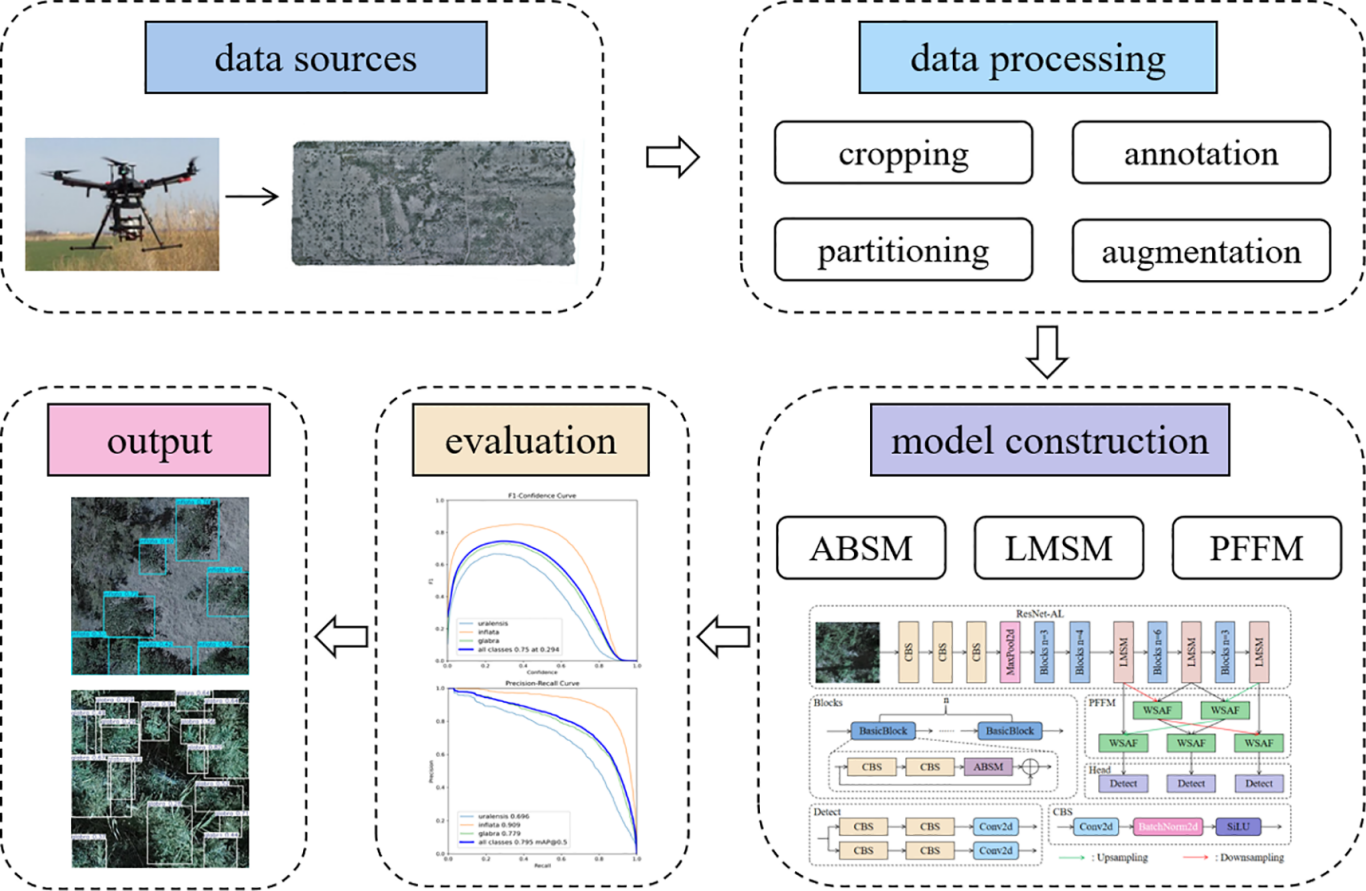
Overall workflow diagram of the study.

The workflow of this study is illustrated in **Figure 1**. First, wild licorice data were collected in Xinjiang using a UAV equipped with a camera and subsequently processed through a series of steps, including data cropping, annotation, partitioning, and augmentation, resulting in a comprehensive wild licorice dataset. Detailed procedures are described in subsections 2.1 and 2.2. Next, the dataset was input into the constructed ALPD-Net model for training; the training environment and parameter settings are detailed in subsections 2.4, while the architecture and module composition of the ALPD-Net model are elaborated in subsection 2.3. Finally, the trained ALPD-Net model was comprehensively evaluated using multiple metrics such as precision and recall. The evaluation metrics are introduced in subsections 2.5, and detailed evaluation and detection results are presented in section 3.

### 2.1 Data sources

In this study, drones were used to capture images of wild G. uralensis, G. glabra, and G. inflata in the wild. The collection sites are located in Xinjiang Uygur Autonomous Region, specifically in Halajun Township, Artux City, Kizilsu Kyrgyz Autonomous Prefecture; Jinhuyang Town, Tumushuke City; and Wensu County, Aksu Prefecture. These areas belong to a temperate continental arid climate, characterized by dry and infrequent rainfall. The vertical distribution range of the three wild licorice species is between 0 and 2000 meters above sea level, and they are suitable for growth in calcareous soils with a PH value of 6.0-8.5 (Bao et al., 2024).

The DJI M300 is a high-performance UAV launched by DJI, offering centimeter-level positioning accuracy, capable of performing precise flight missions. The Zenmuse P1 is a full-frame camera with 45 million pixels, providing high-resolution images (8192×5460) that capture more detailed ground data (Kersten et al., 2022). Therefore, the UAV DJI M300, equipped with the DJI Zenmuse P1 camera, was used to capture images of wild licorice in the wild from July 6 to 8, 2024. The UAV flies at altitudes of 20 meters, 30 meters, and 40 meters, with ground resolutions of 0.24 cm, 0.36 cm, and 0.49 cm, respectively. The UAV flight path was planned with an 80% forward overlap and a 90% side overlap to ensure accurate data calibration and stitching.

### 2.2 Data processing

The images were cropped to a size of 640×640. To ensure the accuracy of the annotations, RTK positioning tools (Stempfhuber and Buchholz, 2012) were used during data collection to mark some samples of the three types of wild licorice on the ground, enabling accurate identification of licorice on the images, thus reducing annotation errors. To ensure the consistency of sample annotations, cropped images are annotated by the same person using the Labelimg tool (https://github.com/tzutalin/labelImg). The label categories for G. uralensis, G. glabra, and G. inflata are uralensis, glabra, and inflata, respectively.

**Figure 2:**
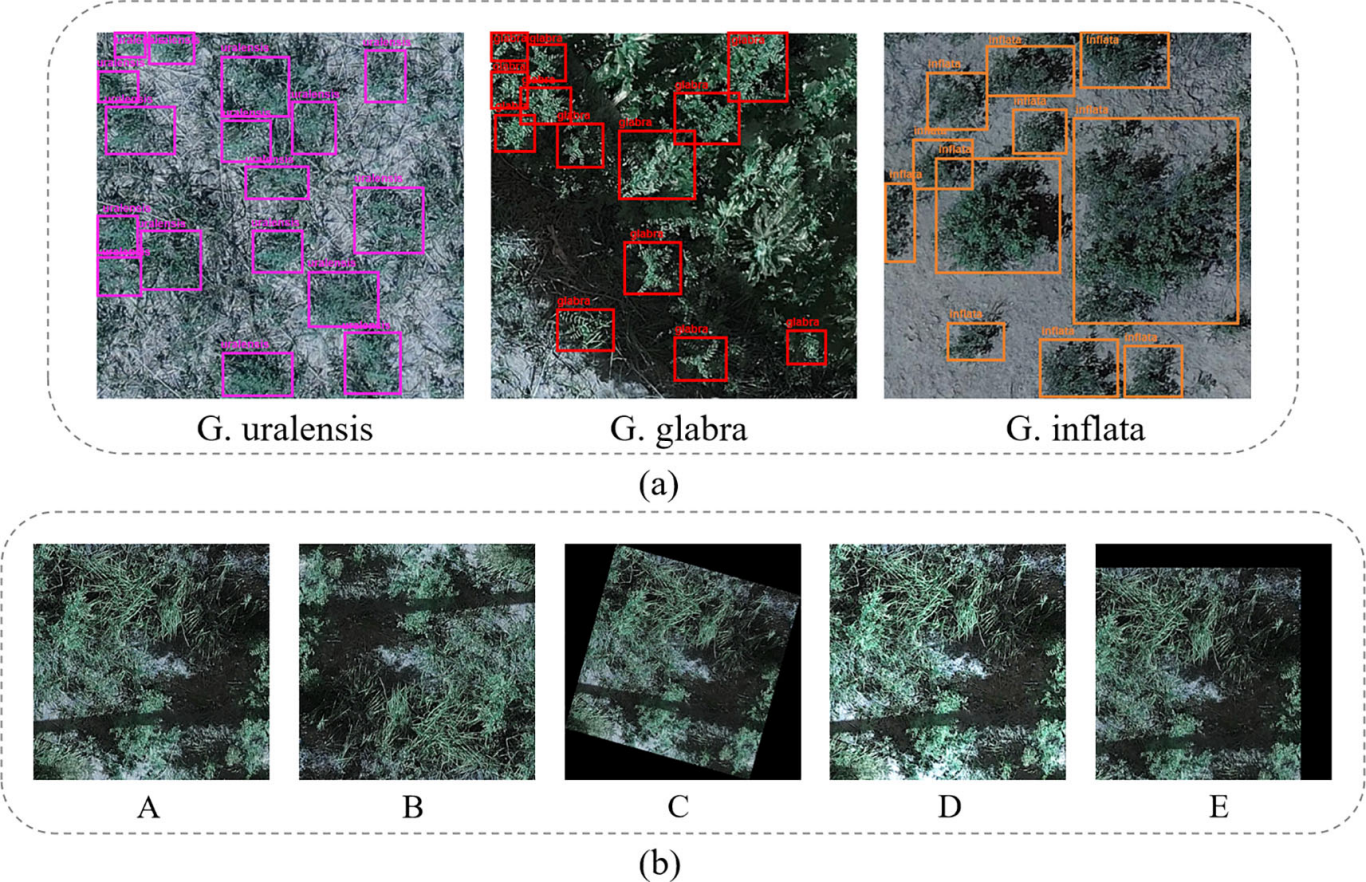
**(a)** shows the annotated image, while **(b)** presents the augmented images. In **(b)**, A represents the original image, B is the mirror-augmented image, C is the rotation-augmented image, D is the brightness augmented image, and E is the translation-augmented image.

To avoid data leakage, the labeled dataset was initially split into training, validation, and test sets in an 8:1:1 ratio. After the split, data augmentation was applied to further enhance the dataset. This includes random rotations, mirroring, brightness transformation, translation, etc (Mumuni and Mumuni, 2022). The data annotation and augmentation process is illustrated in **Figure 2**. A total of 10,260 augmented licorice images are obtained, including 8,208 for the training set, 1,026 for the validation set, and 1,026 for the test set, as shown in **Table 1**.

**Table 1:** Wild licorice dataset.

Type	Training set	Validation set	Test set
G. uralensis	2112	264	264
G. inflata	4320	540	540
G. glabra	1776	222	222

### 2.3 Construction of the ALPD-Net model

**Figure 3:**
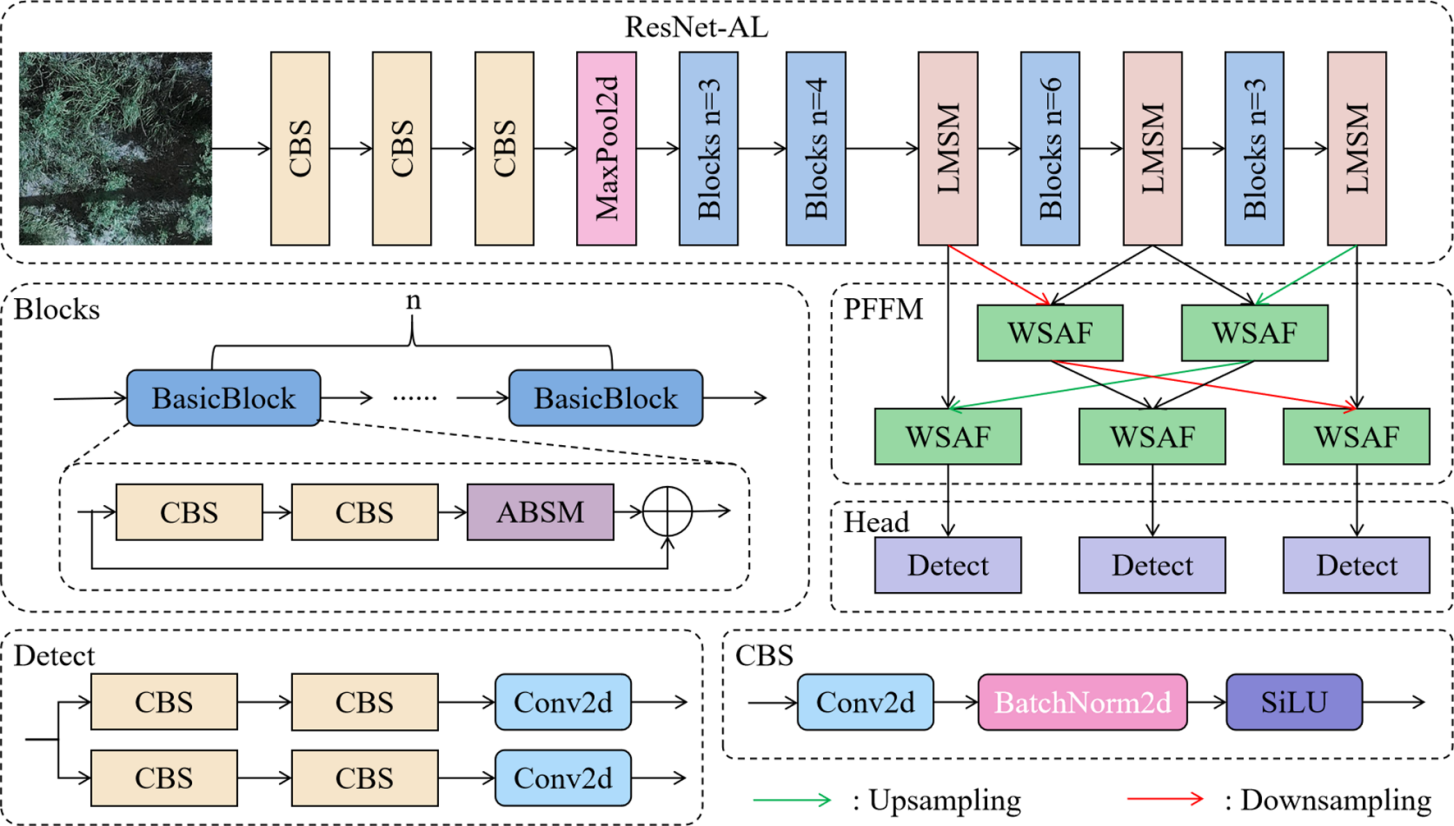
The architecture diagram of the ALPD-Net model.

To improve the performance of the base model ResNet34-D in licorice detection under challenges such as complex backgrounds, multi-scale targets, and high similarity between licorice and backgrounds, ALPD-Net is built, with the model architecture shown in **Figure 3**. The workflow of the model is as follows:

1. The preprocessed licorice images are input into the ResNet-AL backbone network for feature extraction. ResNet-AL is designed based on ResNet34 (Hu et al., 2018), consisting of multiple Blocks modules and LMSM modules stacked together. Each Blocks module is composed of n BasicBlocks, where each BasicBlock contains an ABSM module and a residual connection structure, which not only suppresses interference from complex background information but also alleviates the vanishing and exploding gradient problems in deep neural networks.
2. The multi-scale feature maps output by ResNet-AL are fed into the PFFM. PFFM is a feature fusion structure that uses a progressive strategy to gradually merge adjacent feature layers, enhancing the ability to differentiate between different types of licorice and weeds, further improving the detection performance of ALPD-Net.
3. The fused feature maps are finally input into the Head part to produce the final detection results. The Detect module consists of two parallel branches, each containing two standard convolution layers and one 2D convolution, responsible for generating the bounding boxes, class labels, and confidence scores of the targets.

#### 2.3.1 ABSM

In the wild environment, there are many types of weeds that grow together with licorice, blocking each other, creating a complex background. This background can interfere with the model’s ability to extract licorice features, making it difficult to accurately identify the target and greatly increasing the difficulty of licorice detection. Moreover, during feature extraction, as the network layers increase, the number of channels in the feature maps also gradually increases. Although deep networks can capture more feature information, these feature maps may contain a large amount of redundant and irrelevant information in complex backgrounds (Liu et al., 2024b). This irrelevant information not only increases computational complexity but may also reduce the model’s ability to capture critical licorice information in subsequent processing stages, affecting the model’s accuracy and robustness, and even causing false and missed detections (Zhang et al., 2024).

**Figure 4:**
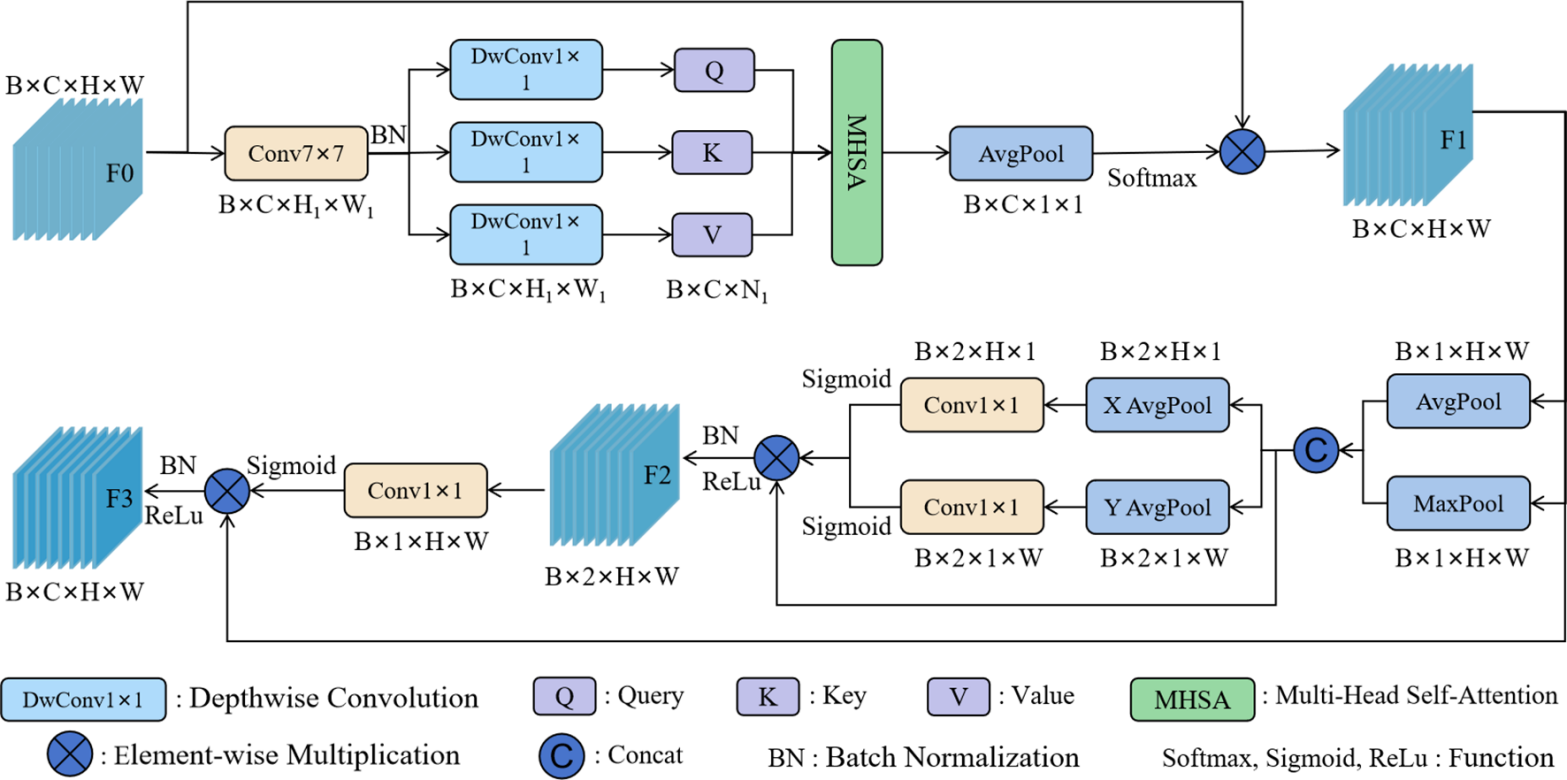
The network structure of the ABSM.

To address the above issues, ABSM was designed to reduce the impact of redundant information on the model’s performance, while enhancing the model’s ability to capture detailed features of licorice in complex backgrounds, thereby improving the model’s detection performance in complex scenes. The network structure of ABSM is shown in **Figure 4**.

ABSM uses a compression strategy on the input feature map by applying a 7×7 convolution to reduce the feature map size. This helps retain key channel information while lowering the computational complexity of the module. The compressed feature map is then input into the Multi-Head Self-Attention (MHSA) mechanism with depthwise convolution, which efficiently computes the similarity between channels while significantly reducing computational costs. This alleviates the semantic differences within channels and aids in accurately filtering out redundant background information. After global average pooling, the channel weights are obtained using the softmax function. The weights are then multiplied by the original feature map to filter out redundant background information, resulting in the channel feature map F1.

The average pooling and max pooling operations are applied to the channels of the channel feature map F1 to obtain the spatial information map of the features. Horizontal and vertical spatial pooling operations are used to accurately capture the coordinate information of the licorice features, and the results are input into the sigmoid function to obtain the coordinate weights. These coordinate weights help the model accurately locate the licorice feature regions in complex backgrounds. The coordinate weights are then multiplied by the spatial feature map to obtain the spatial coordinate feature map F2. F2 contains both the spatial information encoding of the features and the positional information encoding, enhancing the model’s focus on the licorice detail features while suppressing interference from irrelevant information such as complex backgrounds. Through 1×1 convolution and the sigmoid function, the spatial coordinate weights are obtained, and these weights are multiplied by the channel feature map F1 to produce the final feature map, further enhancing the network’s ability to extract and represent features.

#### 2.3.2 LMSM

Wild licorice exhibits significant variation in scale due to the influence of its growth environment. In the detection task of this study, especially when there are large variations in the target scale, the model may fail to extract key information of licorice at all sizes from the feature layers, leading to missed detection (Liu et al., 2024a). Multi-scale feature extraction can help address this challenge of target scale variation to some extent, thereby improving the model’s accuracy, robustness, and generalization capability.

**Figure 5:**
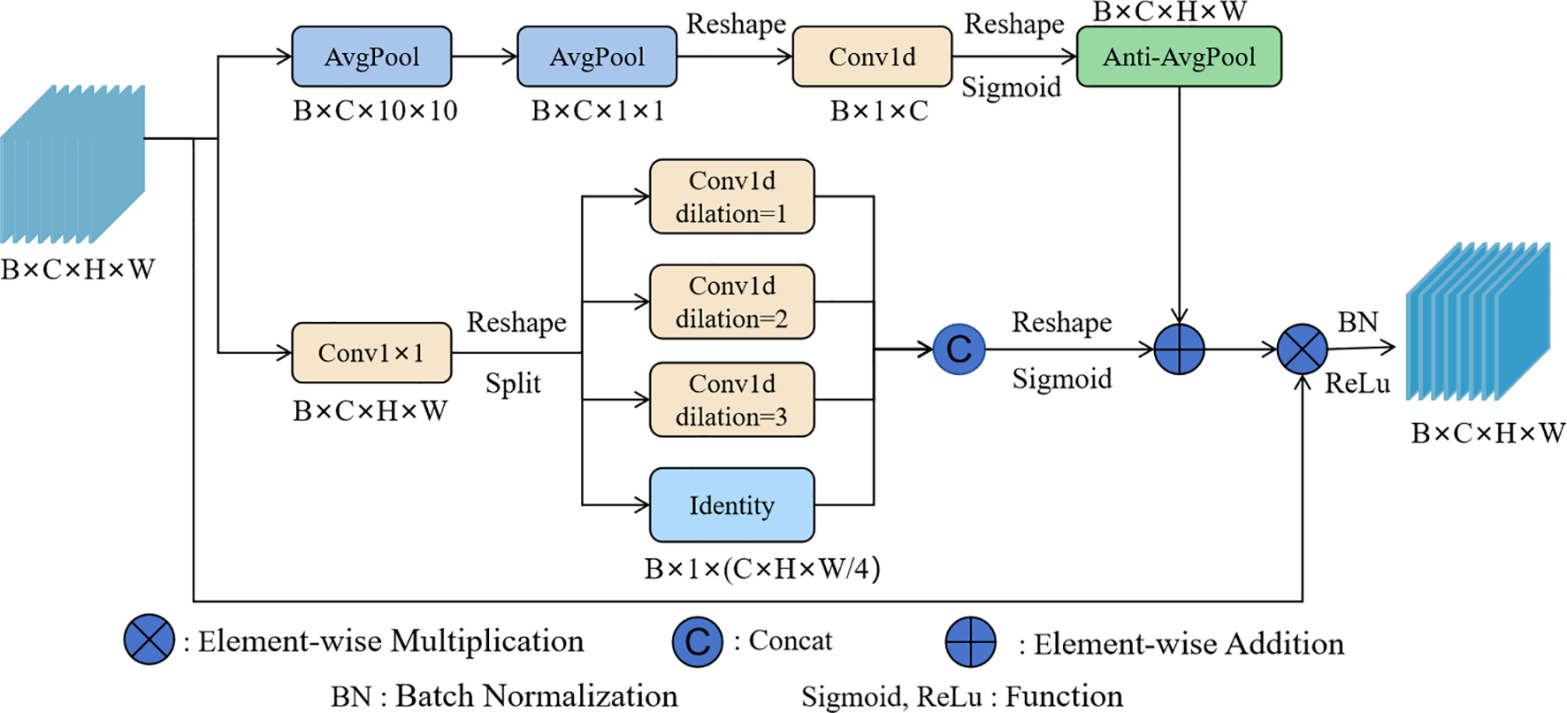
The network structure of the LMSM.

Therefore, LMSM was designed. LMSM can extract licorice features from different scales with minimal computational cost, helping the model quickly and accurately recognize licorice of various sizes, thereby reducing the probability of missed detections. Moreover, single-scale feature extraction may not effectively recognize some or all regions of the licorice. However, LMSM captures licorice features at different scales using multi-scale dilated convolutions. This allows the model to capture both local detail features (such as leaf shape, texture, etc.) and global features (such as the overall structure or distribution pattern of the licorice), better overcoming the problem of weed occlusion and improving the accuracy of licorice detection. The network structure of LMSM is shown in **Figure 5**.

LMSM consists of two branches. The first branch first focuses on the local information in the feature map using local average pooling, and then uses global average pooling to obtain the channel information of the licorice. 1D convolution is used to integrate the channel information, further reducing the impact of irrelevant channel information on licorice recognition, and an anti-pooling operation is applied to restore it to its original size.

The second branch further extracts licorice features through 1×1 convolution and divides them into four parts along the channel dimension. Each part obtains multi-scale features of licorice through dilated convolutions of different scales, and the results are merged using the concat operation, thus further enriching the model’s ability to recognize licorice at different scales. Dilated convolutions expand the receptive field with fewer parameters, capturing a larger range of contextual information. This is particularly helpful when licorice overlaps or is occluded by surrounding weeds or other plants, allowing the model to acquire more information from different scales and enhancing its ability to integrate both detailed and global information.

The features extracted by the two branches are fused, which helps the model better capture the features of licorice at multiple scales while integrating channel information, and also helps to address the occlusion problem to some extent. LMSM has fewer parameters and lower computational cost, improving the model’s detection performance and robustness while maintaining computational efficiency.

#### 2.3.3 PFFM

In the wild licorice detection task, weeds are similar to licorice, and different types of licorice are also very similar to each other, which greatly increases the detection difficulty. Shallow features contain rich detail information, which helps the model recognize subtle differences in the target, while deep features contain rich semantic information, helping the model understand the overall shape and category of the target (Gao et al., 2024). Fusing deep and shallow features helps the model more accurately distinguish between different types of licorice and weeds, thereby improving detection accuracy.

Therefore, the PFFM was designed. Compared to other traditional feature fusion modules, PFFM’s progressive strategy gradually introduces information from more layers, which helps to solve the feature alignment problem to some extent and prevents information loss or incorrect matching. Traditional fusion methods mostly involve simple concatenation or addition, which may not effectively combine the advantages of deep and shallow features, resulting in the model failing to learn useful features (Dai et al., 2024). The WSAF method designed in this study balances the relationship between detail information and semantic information, fully leveraging the strengths of both, and captures long-range dependencies through a MHSA mechanism, reducing the likelihood of misidentifying weeds as licorice, and improving licorice detection performance.

**Figure 6:**
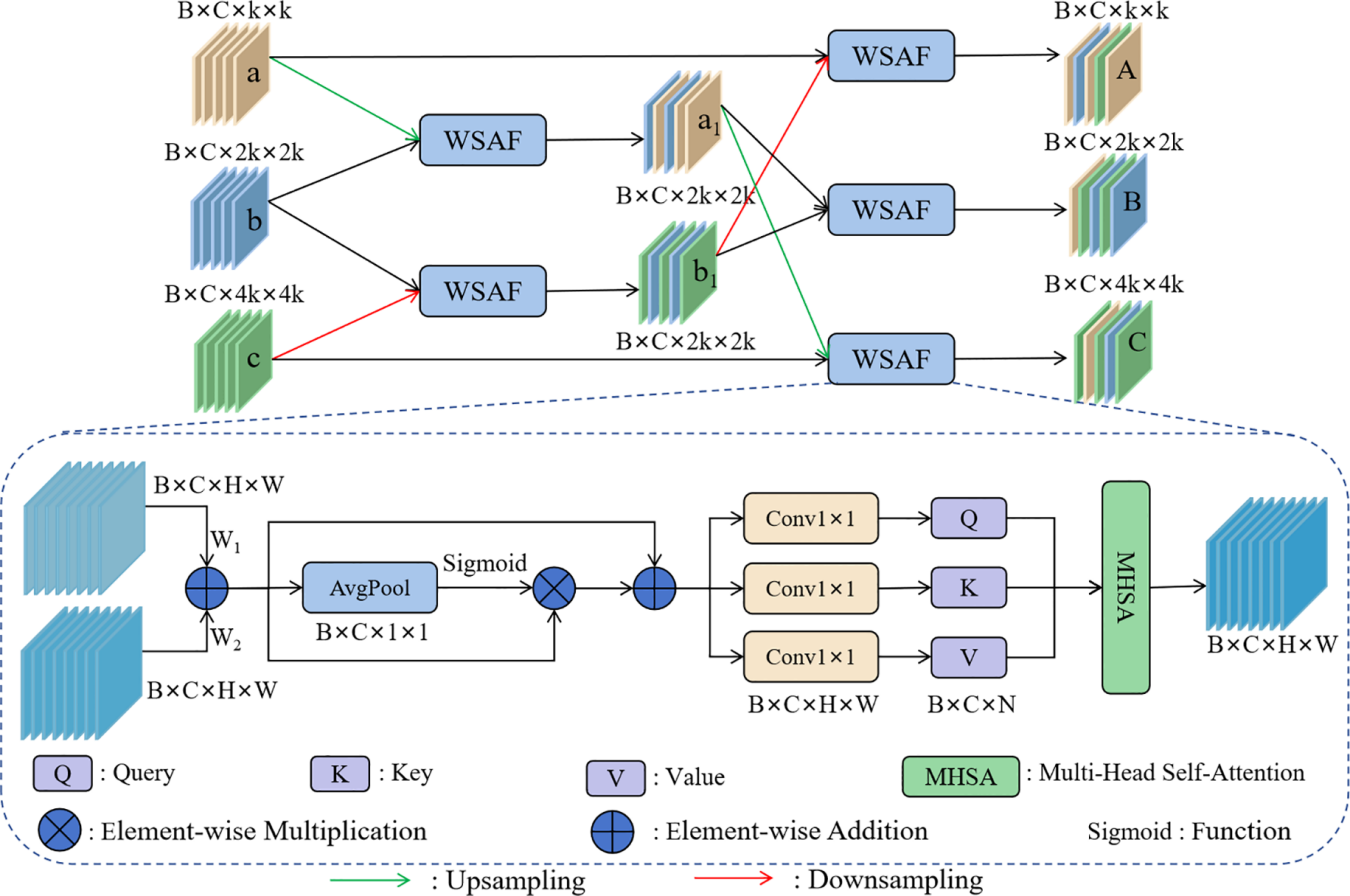
The network structure of the PFFM.

The network structure of PFFM is shown in **Figure 6**. As the network deepens, the size of the feature maps gradually decreases, and the amount of semantic information gradually increases. The adjacent feature maps b and c are initially fused using the WSAF fusion method, and the resulting feature map b1 contains rich detail information. Correspondingly, the feature maps a and b are fused to obtain feature map a1, which contains rich semantic information. Then, a1 is fused again with the feature map c, which itself contains detail information, resulting in feature map C, which contains both rich semantic and detail information.

The process of the progressive fusion strategy is shown in Equations 1–3:


(1) A=WSAF(a,WSAF(b,c))

(2) B=WSAF(WSAF(a,b),WSAF(b,c))

(3) C=WSAF(c,WSAF(a,b))

Where *A*, *B*, and *C* are the feature maps ultimately input into the detection head. By gradually introducing information from adjacent layers, this method better preserves and transmits both detail and semantic information, thus preventing information loss and incorrect matching.

The principle of the WSAF fusion method is shown in **Figure 6**. W_1_ and W_2_ are learnable parameters, which can flexibly adjust the weights of the input feature maps during the training process. After adjustment, the feature maps are fused by addition, effectively integrating detail and semantic information to enhance the model’s performance. The process is shown in Equation 4:


(4) $$F_{1}=F_{01}*W_{1}+F_{02}*W_{2}$$

Where *F*_01_ and *F*_02_ are the feature maps to be fused, and *F*_1_ is the output feature map after addition. Due to the potential generation of redundant information during the fusion process, global average pooling, sigmoid, and multiplication operations are used to filter out the redundant information. To prevent gradient vanishing or gradient explosion during training, the feature map obtained from the above process is added to *F*_1_. Finally, the feature map is input into the multi-head self-attention mechanism, where rich multiperspective information is obtained from different heads. This enhances the model’s ability to capture details and express features, thereby improving the differentiation between different types of licorice and weeds. The process is shown in Equation 5:


(5) $$F_{2}=MHSA(Sigmoid\left(AvgPool\left(F_{1}\right)\right)*F_{1}+F_{1})$$

### 2.4 Experimental environment

The experiments were conducted using PyTorch 2.4.0 as the deep learning framework on an Ubuntu 20.04 operating system. The hardware configuration included an Intel(R) Xeon(R) Gold 5320 CPU and an NVIDIA A30 GPU. The training process of the proposed model was conducted under a well-defined set of hyperparameter configurations to ensure stability and reproducibility. The input images were uniformly resized to 640×640 pixels. The model was trained for 200 epochs with a batch size of 8, and a fixed random seed of 0 was set to maintain consistency across experiments. The Stochastic Gradient Descent (SGD) optimizer was used for model optimization, with an initial learning rate of 0.001, a momentum coefficient of 0.937, and a weight decay of 0.0005 to prevent overfitting (Tian et al., 2023). The training employed an early stopping strategy with a patience of 50 epochs, which means that training would terminate if no improvement was observed over 50 consecutive epochs.

### 2.5 Evaluation metrics

The evaluation metrics selected in this study are: Precision, Recall, mAP50 (mean Average Precision at IoU = 0.50), mAP50-95 (mean Average Precision averaged over IoU thresholds from 0.50 to 0.95 (step=0.05)), and GFLOPs (Giga Floating Point Operations per Second) (Sun et al., 2024; Jegham et al., 2024). Among them, Precision is used to assess the probability that the detected positive samples are true positive samples, while Recall is used to evaluate the proportion of correctly predicted positive samples to all true positive samples. mAP is the average accuracy across multiple categories of the model. mAP50 represents the mAP value at an IOU threshold of 0.5, while mAP50–95 represents the average mAP value over an IOU threshold range of [0.5, 0.95] with a step size of 0.05. GFLOPs is an indicator of computational arithmetic operations, representing the number of floating-point operations that need to be executed per second during the processing. Therefore, the higher the Precision, Recall, mAP50, and mAP50-95, and the lower the GFLOPs, the better the overall performance of the model. The formulas are as follows:


(6) $$Precision=\frac{TP}{TP+FP}$$

(7) $$Recall=\frac{TP}{TP+FN}$$

(8) $$AP=\int_{0}^{1}P\left(R\right)dR$$

(9) $$mAP=\frac{\sum_{i=1}^{n}AP\left(i\right)}{n}$$

(10) $$GFLOPs=O\left(\sum_{i=1}^{n}K_{i}^{2}*C_{i-1}^{2}*C_{i}+\sum_{i=1}^{n}M^{2}*C_{i}\right)$$

In Equations 6, 7, *TP* stands for true positive, *FP* stands for false positive, and *FN* stands for false negative. In Equation 8, *P*(*R*) represents the Precision-Recall (P-R) curve. In Equation 9, *n* is the total number of detection categories. In Equation 10, *O* represents the order of magnitude, *K* is the kernel size, *C* is the number of channels, *M* is the feature map size, and *i* denotes the iteration number.

## 3 Results

### 3.1 Comparison of results before and after model improvements

**Figure 7:**
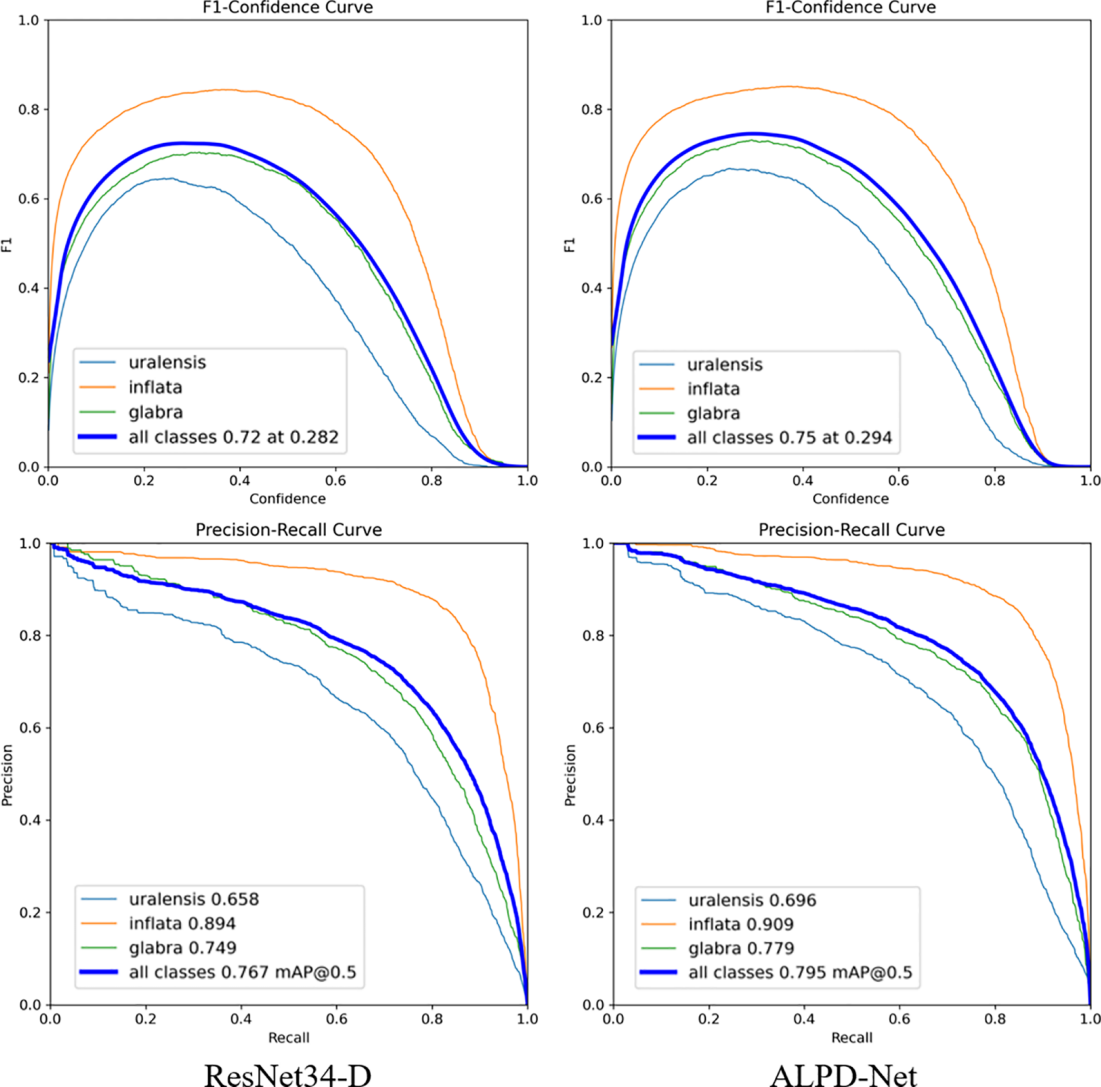
F1-confidence and Precision-Recall curves before and after model improvements: the upper part of the figure shows the F1-confidence curves, while the lower part presents the Precision-Recall curves.

To evaluate the performance changes of the baseline model ResNet34-D before and after improvement, an F1-confidence curve was plotted, as shown in **Figure 7**. The F1 score, as a comprehensive metric, reflects the balance between correctly identifying targets and minimizing both false detection and missed detection. Therefore, the F1-confidence curve effectively illustrates the model’s performance fluctuations under varying confidence thresholds. As observed in the figure, the average F1 score across all categories reaches 0.72 when the confidence threshold is set to 0.282 in the original model. After the model is improved, the average F1 score increases to 0.75 at a higher threshold of 0.294. This shift clearly indicates an overall enhancement in detection performance, with particularly notable improvements in detecting G. uralensis and G. inflata.

The P-R curves before and after the model improvements are shown in **Figure 7**. The P-R curve is used to evaluate the model’s precision performance under varying recall levels, offering a visual representation of the trade-off between correct predictions and both false detection and missed detection. As illustrated in the figure, after the model was improved, the P-R value for G. uralensis increased from 0.658 to 0.696, while that for G. inflata rose from 0.894 to 0.909. Similarly, the P-R value for G. glabra improved from 0.749 to 0.779. Overall, ALPD-Net achieved a higher level of precision and recall across all categories, with the average P-R value increasing to 0.795. This suggests that the refined model achieves a better balance between precision and recall, thereby enhancing the detection performance of ALPD-Net in complex wild field environments.

**Figure 8:**
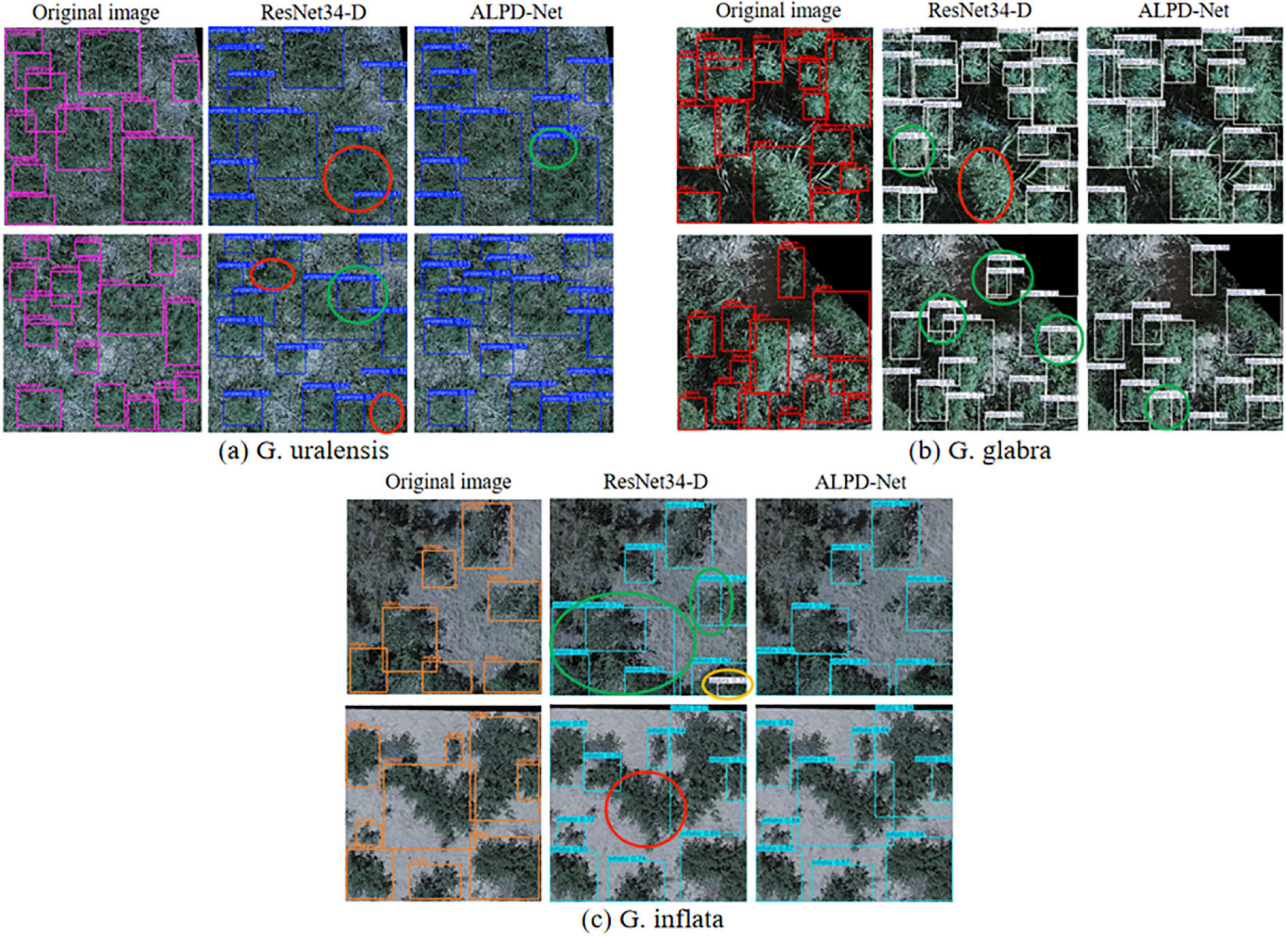
The detection results of wild licorice before and after model improvement are illustrated. Section **(a)** shows images of Glycyrrhiza uralensis, section **(b)** corresponds to Glycyrrhiza glabra, and section **(c)** presents Glycyrrhiza inflata. Red circles indicate missed detections, green circles represent duplicate detections, and orange circles denote false detection.

The detection results of wild licorice before and after model enhancement are shown in **Figure 8**. In the detection results for G. uralensis, it is evident that ResNet34-D exhibits inadequate attention to largescale licorice plants. In the first image, only a portion of the licorice is correctly identified, while the second image demonstrates instances of missed detections in densely populated regions. In contrast, the incorporation of the LMSM multi-scale module significantly improves ALPD-Net’s ability to attend to targets of varying scales, enabling successful detection of large-scale licorice specimens and reducing the incidence of missed detections. In the detection results for G. glabra, ResNet34-D produces redundant detections in the second image. ALPD-Net, on the other hand, benefits from the coordinated interaction among its constituent modules, allowing for more accurate localization and recognition of licorice plants, thereby mitigating the occurrence of such redundant detections to some extent. From the detection results for G. inflata, it is observed that ResNet34-D misclassifies G. inflata as G. glabra in the first image. This type of misclassification is effectively reduced by ALPD-Net due to the presence of the PFFM module, which enhances the model’s capability to distinguish between different licorice species and surrounding weeds, thus improving classification accuracy and reducing false detection.

### 3.2 Ablation experiment

To ensure the effectiveness of the designed modules on the model, ablation experiments were conducted. Based on the baseline model ResNet34-D, the ABSM, LMSM, and PFFM modules were sequentially added to form the models ResNet34-AD, ResNet34-ALD, and ALPD-Net, respectively.

#### 3.2.1 Analysis of the ablation experiment results

The overall evaluation metrics of the model during the improvement process are shown in **Table 2**. Integrating ABSM into the ResNet34’s backbone enhances ResNet34-AD’s performance by minimizing the influence of redundant information and suppressing interference from weed background. This leads to improved detection accuracy in more complex environments, with a 1.3 percentage point increase in mAP50 and a 1 percentage point increase in mAP50-95. This is particularly evident in G. uralensis images, which have more weeds and a more complex background. As shown in **Table 2**, after adding ABSM, ResNet34-AD’s Precision, Recall, mAP50, and mAP50–95 for G. uralensis detection improved by 2.1, 1.3, 2.2, and 1.2 percentage points, respectively, significantly improving the model’s detection accuracy.

**Table 2:** Evaluation metrics of the model during the improvement process.

Models	Class	Precision/%	Recall/%	mAP50/%	mAP50-95/%	GFLOPs
ResNet34-D	G. uralensis	66.3	60.1	65.8	27.3	72.9
	G. glabra	67.7	73.1	74.9	34.3	
	G. inflata	81.0	86.5	89.4	50.0	
	All	71.7	73.2	76.7	37.2	
ResNet34-AD	G. uralensis	68.4	61.4	68.0	28.5	75.6
	G. glabra	67.9	74.1	75.6	34.4	
	G. inflata	81.5	87.3	90.4	51.8	
	All	72.6	74.2	78.0	38.2	
ResNet34-ALD	G. uralensis	68.2	63.6	69.5	28.8	75.7
	G. glabra	68.4	73.6	75.8	35.0	
	G. inflata	82.4	86.7	90.4	51.3	
	All	73.0	74.7	78.6	38.4	
ALPD-Net	G. uralensis	67.9	65.1	69.6	29.0	83.1
	G. glabra	69.9	76.2	77.9	36.5	
	G. inflata	82.1	87.1	90.9	52.0	
	All	73.3	76.1	79.5	39.2	

After further adding LMSM, ResNet34-ALD enhances its focus on the multi-scale features of licorice through the use of multi-scale dilated convolutions. This improvement further boosts Precision and Recall, leading to a 0.6 percentage point increase in mAP50. Since LMSM has very few parameters, the model’s GFLOPs only increase by 0.13%.

After further adding PFFM, its progressive fusion strategy better maintains the transmission of feature information, and the WSAF fusion method effectively integrates detail and semantic information. This significantly improves ALPD-Net’s capacity to differentiate between various types of licorice and weeds, resulting in a further increase of 1.4 percentage points in Recall, with mAP50 reaching 79.5% and mAP5095 reaching 39.2%. This is more pronounced in the detection of G. glabra, where after adding PFFM, the model’s Recall for G. glabra detection increases by 2.6%, reducing the probability of misidentifying G. glabra as other types of licorice or weeds. The Recall for G. uralensis and G. inflata also increases by 1.5 and 0.4 percentage points, respectively.

The improved model shows a significant increase in precision and recall for licorice detection, reducing the probability of false and missed detections. This indicates that ALPD-Net can more effectively identify licorice, leading to better performance in wild licorice detection tasks.

#### 3.2.2 Analysis of the model’s result visualization

**Figure 9:**
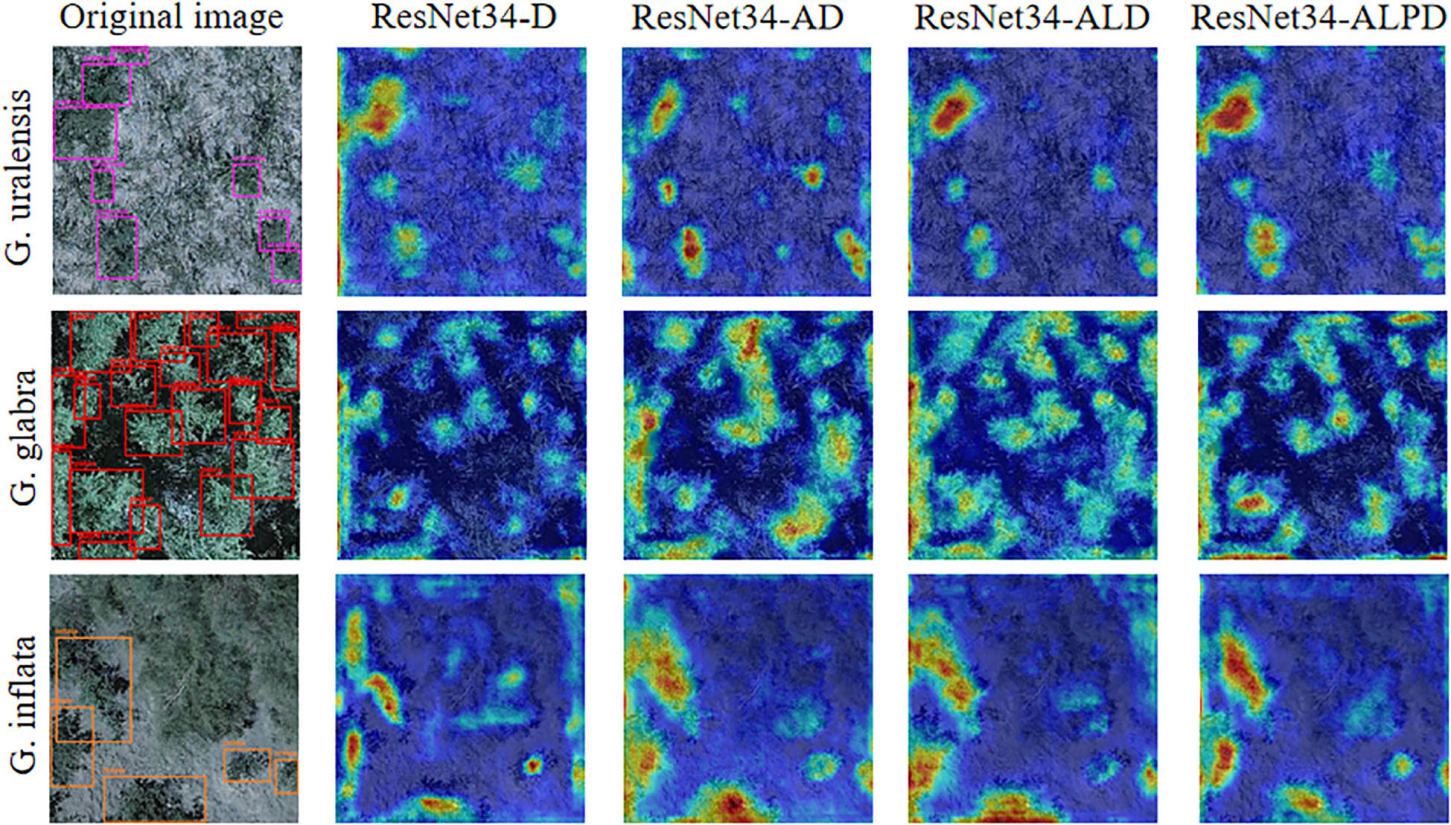
Heatmap visualization of detection results for G. uralensis, G. glabra, and G. inflata during the model improvement process.

To better illustrate how different modules affect the model’s learning ability during the improvement process, Grad-CAM visualization is employed to compare the features extracted by the model, as shown in **Figure 9**.

From the heatmaps of the three types of licorice on ResNet34-D in **Figure 9**, it can be seen that the model does not pay enough attention to licorice features, and even in the G. inflata heatmap, there is still attention to the weed background information. After adding ABSM, the feature extraction ability of ResNet34-AD in complex backgrounds is enhanced, making the extraction of licorice detail features more complete, and the error in extracting weed background information in the G. inflata heatmap is significantly improved. After adding LMSM, from the licorice heatmap of ResNet34-ALD, it can be seen that, regardless of whether the licorice is large or small in size, the model can accurately extract licorice features, thanks to the multi-scale dilated convolution. After adding PFFM, the semantic and detail information are fully fused, enabling the model to precisely distinguish weeds from different types of licorice, making the contours of the licorice feature areas in the heatmap clearer.

### 3.3 Comparison with mainstream multi-scale modules

The LMSM module proposed in this paper is compared with mainstream multi-scale modules, including Multi-Scale Convolutional Attention (MSCA) (Ding, 2023), Spatial Pyramid Pooling - Fast (SPPF) (Jocher et al., 2023), and Multi-Scale Dilated Attention (MSDA) (Jiao et al., 2023), to demonstrate its effectiveness in multi-scale feature extraction under wild background conditions. All experiments were conducted based on ResNet34-AD. As shown in **Table 3**, the mAP@50 scores after integrating MSCA, SPPF, and MSDA are 78.2%, 78.1%, and 78.2% respectively, which are slightly inferior to the result achieved with the LMSM module. MSCA captures multi-scale information through multi-branch depth-wise strip convolutions; SPPF enhances multi-scale feature extraction using max pooling operations; and although MSDA utilizes multi-scale dilated convolutions, its self-attention mechanism requires significant computational resources. In contrast, LMSM integrates multi-scale dilated convolutions with 1D convolutions, enabling the model to better focus on licorice roots of varying sizes with minimal computational cost. As also shown in the table, the computational load with LMSM is only 75.7%, which is lower than that of MSCA (76.0%), SPPF (76.7%), and MSDA (77.6%).

**Table 3:** Comparison results between LMSM and mainstream multi-scale modules.

Models	Precision/%	Recall/%	mAP50/%	mAP50-95/%	GFLOPs
ResNet34-AD+MSCA	73.5	74.1	78.2	38.6	76.0
ResNet34-AD+SPPF	71.5	74.6	78.1	38.1	76.7
ResNet34-AD+MSDA	73.7	74.6	78.2	38.4	77.6
ResNet34-ALD(+LMSM)	73.0	74.7	78.6	38.4	75.7

### 3.4 Comparison with mainstream fusion modules

To verify the effectiveness of the proposed WSAF in feature fusion, it is compared with several mainstream fusion modules, including the Cross-Attention Fusion Module (CAFM) (Zhou et al., 2023), Efficient Feature Fusion (EFF) (Li et al., 2023), Dynamic Feature Fusion (DFF) (Yang et al., 2024a), and iterative Attentional Feature Fusion (iAFF) (Dai et al., 2021). All comparative experiments were conducted based on ResNet34-ALD, and the results are shown in **Table 4**.

**Table 4:** The impact of WSAF and mainstream fusion modules on licorice detection.

Models	Precision/%	Recall/%	mAP50/%	mAP50-95/%	GFLOPs
ResNet34-ALD+CAFM	73.3	76.1	79.3	38.9	78.5
ResNet34-ALD+EFF	72.1	76.0	78.6	38.9	78.6
ResNet34-ALD+DFF	71.8	76.7	78.7	39.0	80.6
ResNet34-ALD+iAFF	73.2	76.3	78.9	39.2	80.7
ALPD-Net(+WSAF)	73.3	76.1	79.5	39.2	83.1

CAFM enhances information interaction between different feature layers through cross-channel attention and spatial attention mechanisms, thereby improving the representation of target features. This method demonstrates stable performance on the licorice image dataset, achieving a mAP50 of 79.3%, slightly lower than WSAF. The EFF module integrates multi-scale features using multiple attention mechanisms; however, it suffers from information loss when handling object boundary details, resulting in a mAP50 of only 78.6%. DFF adaptively fuses local features based on global information, but its discriminative power is limited when faced with highly similar background regions, yielding a mAP50 of 78.7%. The iAFF module enhances the robustness of initial fusion results through iterative application of attention mechanisms, which helps alleviate feature misalignment issues to some extent, reaching a mAP50 of 78.9%. In contrast, WSAF combines adaptive weighting with multi-head self-attention mechanisms to better balance detail and semantic information during fusion. This enhances the model’s discriminative ability for licorice targets under complex backgrounds, ultimately achieving a mAP50 of 79.5%, outperforming all aforementioned comparison modules and demonstrating superior feature fusion and detection performance.

### 3.5 Comparison with mainstream backbone networks

To better demonstrate the excellent feature extraction ability of the backbone network ResNet-AL in more complex backgrounds, a comparison is made between the mainstream backbone networks and ResNet-AL under the same dataset, experimental parameters, and detection head. The comparison results are shown in **Table 5**.

**Table 5:** Comparison results of ResNet-AL with mainstream backbone networks.

Backbone	Precision/%	Recall/%	mAP50/%	mAP50-95/%	GFLOPs
ResNet18	71.0	73.7	76.4	36.4	42.6
ResNet34	71.7	73.2	76.7	37.2	72.9
ResNet50	69.5	74.0	75.8	36.2	183.1
ResNet101	71.8	71.4	75.9	36.5	244.1
SENet	71.0	73.1	76.7	36.8	68.7
DenseNet121	73.6	72.2	76.7	35.8	179.2
ConvNeXt V2	68.0	70.1	72.7	34.6	31.0
RepViT	67.8	70.6	74.1	35.1	46.8
EfficientViT	69.4	70.1	74.2	34.8	44.3
Swin Transformer	68.0	68.5	72.0	32.1	90.4
Ours(ResNet-AL)	73.0	74.7	78.6	38.4	75.7

ResNet18, ResNet34, ResNet50, ResNet101, and SENet (Hu et al., 2018) are convolutional neural networks with residual connections (He et al., 2016; Alzubaidi et al., 2021). They perform well in simple backgrounds, but their feature extraction ability may not be fully utilized when handling more complex backgrounds or when the background and target are highly similar, which affects detection accuracy. However, ABSM in ResNet-AL suppresses the interference of complex background information, making it superior to the aforementioned backbone networks in terms of Precision, Recall, mAP50, and mAP50-95. DenseNet121 (Huang et al., 2017) is a densely connected convolutional neural network with better feature extraction ability in complex backgrounds, and its Precision is 0.6 percentage points higher than that of ResNet-AL in this paper. However, its ability to distinguish between different types of licorice and weeds is weak, resulting in a lower recall rate. Additionally, DesNet121 has a large GFLOPs, 2.4 times that of ResNet-AL. ConvNeXt V2 (Woo et al., 2023) performs excellently on standard datasets, but the cluttered information in wild backgrounds may interfere with the model’s extraction of fine-grained features. Its Precision and Recall only reach 68.0% and 70.1%, making its detection performance in wild scenarios inferior to ResNet-AL. RepViT (Wang et al., 2024a), EfficientViT (Liu et al., 2023), and Swin Transformer (Liu et al., 2021) are Transformer-based models that perform well in handling long-range dependencies and complex background information. However, wild environments are more complex, and various factors interfere with each other, which may result in these models having difficulty handling local details or separating targets from the background, leading to suboptimal detection performance.

The experimental results in **Table 5** provide a clear justification for selecting ResNet34 as the backbone network in this study. As shown, ResNet34, SENet, and DenseNet121 all achieve an identical mAP50 of 76.7%. However, ResNet34 demonstrates superior performance in mAP50–95, reflecting enhanced accuracy under more stringent evaluation thresholds. Furthermore, although ResNet34 incurs slightly higher GFLOPs than SENet, it remains significantly more computationally efficient than DenseNet121. These findings indicate that ResNet34 strikes an effective balance between detection accuracy and computational cost, making it a well-suited foundational backbone for the proposed model.

In summary, ResNet-AL effectively reduces the impact of complex wild background information, improving the capacity to extract detailed licorice features. It achieves a mAP50 of 78.6%, a mAP50–95 of 38.4%, and a significantly lower GFLOPs compared to ResNet50, ResNet101, DenseNet121, and Swin Transformer, leading to superior detection performance.

### 3.6 Comparison with classic models

This paper compares the model ALPD-Net with mainstream object detection models YOLOv5x (Cheng et al., 2024), YOLOv6x (Li et al., 2022), YOLOv8x (Jocher et al., 2023), YOLOv9e (Wang et al., 2024d), YOLOv10x (Wang et al., 2024b), YOLOv11x (Khanam and Hussain, 2024), MAF-YOLOm (Yang et al., 2024c), Hyper-YOLOx (Feng et al., 2025), and RT-DETR (Zhao et al., 2024). All the models are single-stage object detection models. Due to the many sizes of the comparison models, the largest size model is selected for training comparison to highlight the excellent licorice detection performance of ALPD-Net. The dataset, experimental configuration, and experimental parameters during the training process remain consistent. The comparison results are shown in **Table 6**.

**Table 6:** Comparison results of different models.

Models	Precision/%	Recall/%	mAP50/%	mAP50-95/%	GFLOPs
YOLOv5x	71.1	74.6	77.3	38.1	246.0
YOLOv6x	71.9	73.5	77.2	38.4	610.3
YOLOv8x	71.4	74.8	77.7	38.4	257.4
YOLOv9e	71.8	77.4	79.1	40.2	189.1
YOLOv10x	72.9	72.3	77.0	38.4	169.8
YOLOv11x	71.2	73.8	78.3	39.2	194.4
MAF-YOLOm	—	—	76.4	38.2	76.7
Hyper-YOLOx	70.7	74.9	77.7	38.9	328.8
RT-DETR	60.4	60.0	59.6	16.5	222.5
Ours(ResNet34-ALPD)	73.3	76.1	79.5	39.2	83.1

YOLOv5x, YOLOv6x, YOLOv8x, YOLOv9e, YOLOv10x, and YOLOv11x are mainstream versions of the YOLO series, with each version showing varying degrees of performance improvement. YOLOv5x focuses on the efficiency of the model, YOLOv6x further optimizes the network structure and enhances multi-scale detection capabilities, while YOLOv8x and YOLOv9e innovate the model to improve robustness in complex scenarios. YOLOv10x and YOLOv11x optimize the balance between inference speed and accuracy, making them suitable for applications that require high performance and precision. Compared to the above models, ResNet34-ALPD outperforms them in mAP50 by 2.2, 2.3, 1.8, 0.4, 2.5, and 1.2 percentage points, respectively. Among them, YOLOv9e’s overall performance is comparable to ResNet34ALPD, and its Recall and mAP50–95 are even 1.3 and 1 percentage points higher than those of this paper. However, it is worth noting that the GFLOPs of ResNet34-ALPD are smaller, accounting for 33.8%, 13.7%, 32.3%, 43.9%, 48.9%, and 42.7% of theirs, respectively. ResNet34-ALPD consumes fewer computational resources while achieving higher detection performance.

MAF-YOLOm model contains a multi-branch auxiliary feature pyramid network that facilitates the fusion of shallow feature information while achieving a multi-scale receptive wild. Although its GFLOPs are slightly lower than ResNet34-ALPD, ResNet34-ALPD achieves 3.1 and 1 percentage points higher in mAP50 and mAP50-95, respectively. This enhancement is due to the ABSM, which minimizes background interference during feature extraction, enabling the fusion module to process feature maps containing rich details and semantic information of licorice targets, thus boosting ResNet34-ALPD’s detection accuracy. The Hyper-YOLOx model integrates hypergraph computation to capture complex higher-order correlations between visual features, performing excellently on the COCO dataset, but its detection performance declines in complex wild scenarios, with mAP50 being 1.8 percentage points lower than the model in this paper. RT-DETR is an end-to-end detection framework based on Transformer, capable of real-time detection. In challenging wild environments, where distinguishing various types of licorice is crucial, the need for real-time processing may restrict the model’s complexity, hindering its ability to handle such complex scenarios and resulting in suboptimal performance in wild licorice detection.

**Figure 10:**
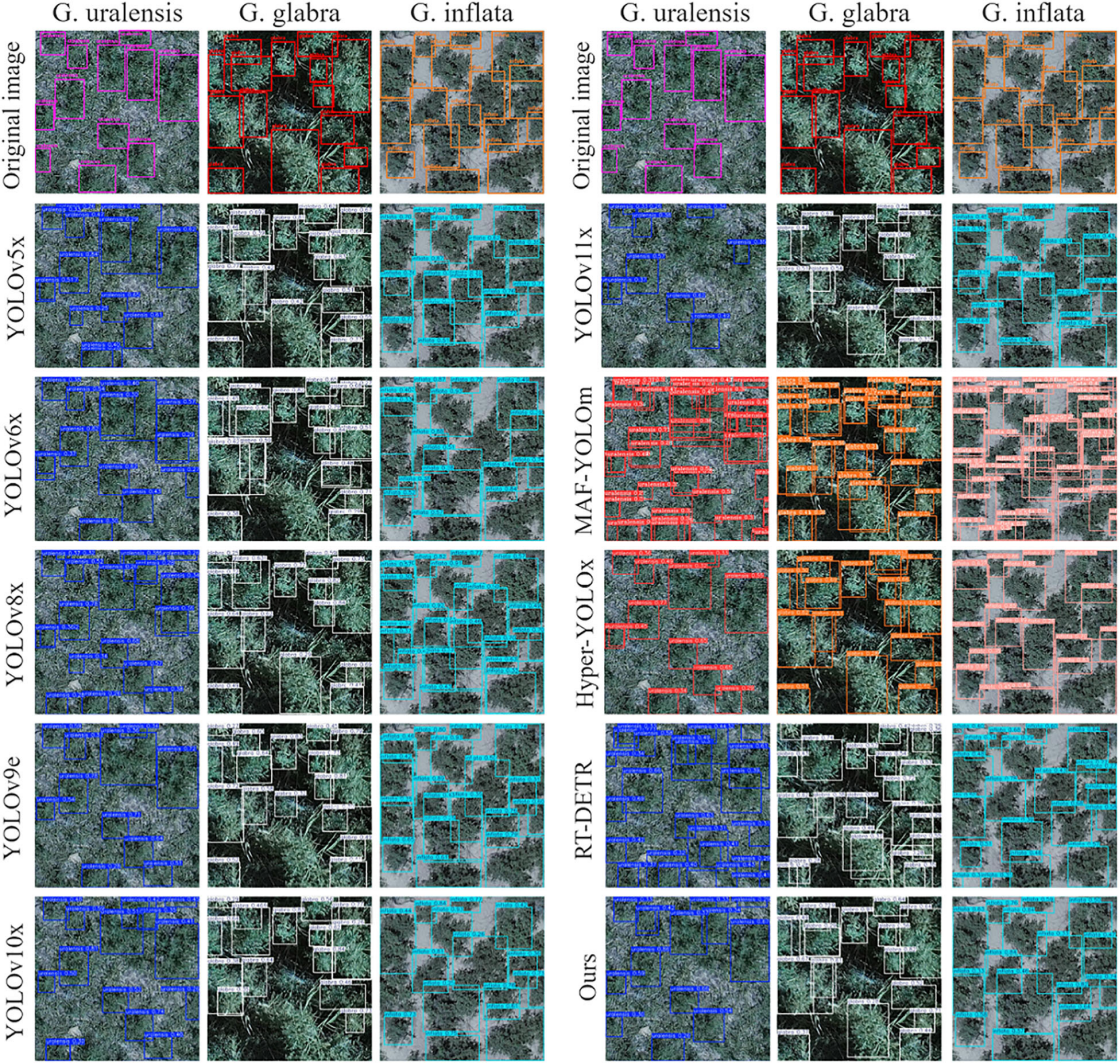
The detection results of ALPD-Net and mainstream models on G. uralensis, G. glabra, and G. inflata.

The licorice detection results of the comparison models are shown in **Figure 10**. In images of G. uralensis and G. inflata, the comparison models generally experience false and missed detections due to the presence of complex backgrounds and the high similarity between various types of licorice and weeds. On the other hand, the ABSM module in ResNet34-ALPD improves the capability to capture detailed features of licorice in complex environments, while PFFM, by merging detailed and semantic information, enhances ResNet34-ALPD’s capability to differentiate between licorice and weeds, effectively reducing both false and missed detections. In G. glabra images, models like YOLOv6x, YOLOv9e, and YOLOv10x fail to detect large-scale licorice. However, ResNet34-ALPD performs well in detecting large-scale licorice, mainly due to the outstanding multi-scale feature extraction ability of LMSM, which significantly lowers the rate of missed detection.

Overall, the ResNet34-ALPD model demonstrates both high detection accuracy and low computational cost, showcasing strong licorice detection capabilities in complex wild scenarios.

## 4 Discussions and conclusions

This study constructed a novel wild licorice dataset and conducted an in-depth analysis of the improvement process of ALPD-Net, comparing it with single-stage object detection models. The experimental results show that the improved ALPD-Net achieved 73.3% in Precision, 76.1% in Recall, 79.5% in mAP50, and 39.2% in mAP50-95. These results consistently outperformed other object detection models across multiple evaluation metrics, demonstrating ALPD-Net’s strong capability in detecting licorice targets in complex wild environments.

The wild scenarios addressed in this study differ from single, fixed complex environments. The three types of wild licorice grow in distinct habitats, resulting in diverse and highly complex visual scenes. Additionally, wild environments often contain dense weed coverage, with significant occlusion between licorice and surrounding vegetation, which adds substantial difficulty to accurate licorice detection (Wang et al., 2021). To address challenges posed by complex backgrounds, several researchers have proposed architectural improvements. For instance, Sun et al. (2024) incorporated the Simple Attention Module (SimAM) to enhance feature extraction of tobacco pests in cluttered scenes. However, their background complexity was relatively limited, with minimal interference, potentially weakening the model’s ability to capture fine-grained details in more intricate natural environments. Similarly, Yang et al. (2024b) employed cross-modal transformer attention to improve feature extraction across channel and spatial dimensions under complex backgrounds, achieving more accurate detection. Nonetheless, their method did not adequately address the presence of redundant and irrelevant information. In contrast, the ABSM module proposed in this study encodes spatial positional information in the feature maps to mitigate the influence of redundant data on model performance, while simultaneously enhancing the ALPD-Net’s ability to capture fine-grained features of licorice in highly complex wild scenes.

Wild licorice exhibits considerable variation in morphology and target size across natural environments. Under such conditions, a single feature layer may struggle to effectively capture key information for licorice targets of different scales (Wu et al., 2025). To address the challenge of multi-scale object detection, Ding (2023) designed the MSCA module, and Jiao et al. (2023) proposed the MSDA module. While these approaches have improved detection performance for multi-scale objects to some extent, they often come with significant computational overhead, which adversely affects overall inference efficiency. In contrast to these methods, the LMSM module proposed in this study adopts a multi-scale dilated convolution structure. By introducing only a minimal number of parameters, it effectively enhances ALPD-Net’s ability to perceive licorice targets with large scale variations, achieving a better balance between accuracy and efficiency. Finally, the PFFM module was designed in this paper. Since some weeds in the wild are very similar to licorice, there is a possibility of confusing licorice with background weeds. The PFFM module adopts a progressive strategy and uses the WSAF fusion method to fully integrate detail and semantic information. Compared to traditional feature fusion modules introduced in subsection 3.4, such as CAFM, EFF, and others, the PFFM module not only effectively addresses the issue of incorrect information matching but also significantly enhances the model’s ability to distinguish between licorice and weeds. Under the combined effect of these three modules, ALPD-Net demonstrates excellent licorice detection capability in challenging wild scenarios.

Although ALPD-Net demonstrates strong accuracy and efficiency in detecting wild licorice under natural field conditions, several limitations remain. First, the dataset utilized in this study was collected exclusively from three locations in Xinjiang and focuses solely on licorice during the flowering stage. Given the diversity and complexity of field environments, licorice of the same species may exhibit varying visual characteristics across different regions, climatic conditions, and phenological stages. This constraint may limit the model’s generalization capability and robustness in broader applications. To address this, future research should aim to build a more diverse and representative dataset by including samples from multiple geographic regions and various growth stages, thereby enabling a more comprehensive evaluation of the model’s cross-regional and temporal generalizability. Second, although the model exhibits high detection efficiency, it currently lacks real-time processing capability—a critical requirement for practical deployment on UAV platforms. To improve real-time applicability, future efforts should explore model compression techniques such as pruning and quantization, along with hardware acceleration strategies. These enhancements would help reduce computational complexity while preserving accuracy, facilitating deployment on resource-constrained embedded systems for real-time field monitoring.

## Data Availability

The original contributions presented in the study are included in the article/supplementary material. Further inquiries can be directed to the corresponding authors.
